# Dorsal Vagal Complex Modulates Neurogenic Airway Inflammation in a Guinea Pig Model With Esophageal Perfusion of HCl

**DOI:** 10.3389/fphys.2018.00536

**Published:** 2018-05-15

**Authors:** Zhe Chen, Lejia Sun, Hui Chen, Dachuan Gu, Weitao Zhang, Zifeng Yang, Tao Peng, Rong Dong, Kefang Lai

**Affiliations:** ^1^The First People’s Hospital of Kunshan, Jiangsu University, Kunshan, China; ^2^State Key Laboratory of Respiratory Disease, Guangzhou Institute of Respiratory Disease, The First Affiliated Hospital of Guangzhou Medical University, Guangzhou, China; ^3^Department of Hepatobiliary Surgery, Peking Union Medical College, Chinese Academy of Medical Sciences (CAMS), Beijing, China; ^4^ICU, First Affiliated Hospital of Soochow University, Suzhou, China; ^5^Department of Cardiothoracic Surgery, Fu Wai Hospital, Beijing, China; ^6^Department of Urology, Zhongshan Hospital, Fudan University, Shanghai, China; ^7^Department of Physiology, Medical School of Southeast University, Nanjing, China

**Keywords:** gastroesophageal reflux, dorsal vagal complex, substance P, neurogenic inflammation, chronic cough

## Abstract

Neurogenic airway inflammation in chronic cough and bronchial asthma related to gastroesophageal reflux (GER) is involved in the esophageal–bronchial reflex, but it is unclear whether this reflex is mediated by central neurons. This study aimed to investigate the regulatory effects of the dorsal vagal complex (DVC) on airway inflammation induced by the esophageal perfusion of hydrochloric acid (HCl) following the microinjection of nuclei in the DVC in guinea pigs. Airway inflammation was evaluated by measuring the extravasation of Evans blue dye (EBD) and substance P (SP) expression in the airway. Neuronal activity was indicated by Fos expression in the DVC. The neural pathways from the lower esophagus to the DVC and the DVC to the airway were identified using DiI tracing and pseudorabies virus Bartha (PRV-Bartha) retrograde tracing, respectively. HCl perfusion significantly increased plasma extravasation, SP expression in the trachea, and the expression of SP and Fos in the medulla oblongata nuclei, including the nucleus of the solitary tract (NTS) and the dorsal motor nucleus of the vagus (DMV). The microinjection of glutamic acid (Glu) or exogenous SP to enhance neuronal activity in the DVC significantly potentiated plasma extravasation and SP release induced by intra-esophageal perfusion. The microinjection of γ-aminobutyric acid (GABA), lidocaine to inhibit neuronal activity or anti-SP serum in the DVC alleviated plasma extravasation and SP release. In conclusion, airway inflammation induced by the esophageal perfusion of HCl is regulated by DVC. This study provides new insight for the mechanism of airway neurogenic inflammation related to GER.

## Introduction

Gastroesophageal reflux (GER) is one of the most common causes of chronic cough and is associated with severe asthma (Irwin et al., 1981, 1990; Klauser et al., 1990; Mello et al., 1996; Harding and Richter, 1997; Leggett et al., 2005; Lai et al., 2013); however, its pathogenesis is poorly understood. It was traditionally thought that aspiration leads to chronic cough and even asthma(Ayres and Miles, 1996; Canning and Mazzone, 2003), but a study shows proximal GER and microaspiration into the airways have limited roles in provoking chronic cough (Decalmer et al., 2012), and increasing evidence indicates that neurogenic inflammation induced by the esophageal-bronchial reflex plays an important role (Stein, 2003; Kollarik and Brozmanova, 2009). Single acid perfusion into the distal esophagus induces microvascular leakage, which is suppressed by a neurokinin 1 receptor (NK1R) antagonist or by cutting the bilateral vagus, indicating that acid stimulation leads to the release of substance P (SP) and neurogenic inflammation (Hamamoto et al., 1997). Distal esophageal acid perfusion in rabbits decreases airway resistance and lung compliance, which is preventable by the administration of NK1R and a neurokinin 2 receptor (NK2R) antagonist (Gallelli et al., 2003). Neurokinin B (NKB) also causes airway microvascular leakage in guinea pigs (Daoui et al., 2002). Therefore, experimental evidence indicates the importance of neurogenic inflammation in the airway induced by GER via the esophageal-bronchial reflex. In a previous study, we established a guinea pig model with GER by performing repeated esophageal HCl perfusion and observed increased plasma leakage and neuropeptides, such as SP, neurokinin A (NKA), and NKB in lung tissues (Liu et al., 2013), and also the airway hyperresponsiveness and remodeling could be induced (Cheng et al., 2014). Irwin et al. (1993) proposed that the activation of mucous membrane receptors, rather than aspiration, is the main mechanism underlying cough. Cough severity parallels the severity of acid reflux (Wunderlich and Murray, 2003). Approximately 78% of patients with acid reflux experience cough, and continuous reflux lasting more than 5 min may lead to paroxysms of coughing. In addition, acid perfusion in GER disease (GERD) patients also worsens their cough (Ing et al., 1992; Ing and Ngu, 1999). These studies suggest that cough is closely associated with GER, and airway neurogenic inflammation could be induced by acid stimulation in the esophagus. Besides microaspiration and reflux, the sensitized esophageal–bronchial neuronal pathway is also important (Houghton et al., 2016). However, whether the esophageal-bronchial reflex and neurogenic inflammation of the airway are involved in the modulation of central nervous system (CNS) remains unclear.

The primary neurogenic inflammatory mediators include SP, calcitonin gene-related peptide (CGRP), NKA, and NKB, which are released by sensory nerve terminals. These mediators cause vasodilatation, plasma leakage, and cough. In a previous study, we found that SP expression in the nodose ganglion was increased in a guinea pig model of GER (Liu et al., 2013). The nodose ganglion is the first relay neuron of the afferent sensory nerves, and the nucleus of the solitary tract (NTS) comprises secondary CNS neurons from the afferent sensory nerves. Studies have shown that airway inflammation could affect the CNS neurons thereby modifying the airway response. The μ-Opioid receptors in the CNS could modulate the psychological stress-induced aggravation of allergic airway inflammation (Okuyama et al., 2010). The allergic airway inflammation may cause a hyperexcitable state of the airway-related vagal preganglionic neurons, and centrally mediated airway hyperreactivity (Wilson et al., 2007). The parasympathetic, sympathetic, and sensory nerves innervate airways and adjust airway reflex via receptors and neurotransmitters (Kc and Martin, 2010; Audrit et al., 2017). It is unclear whether the central nuclei are involved in or modulate the release of inflammatory mediators in the lung. We hypothesized that when acid stimulates the esophagus, sensory information reaches the medulla oblongata and induces changes in the activity and transmitter expression of neurons. These effects subsequently induce or intensify airway inflammation via efferent nerves, potentially constituting an important mechanism underlying chronic refractory cough and severe asthma associated with GER. This study aimed to observe the activity and neurotransmitter expression of dorsal vagal complex (DVC) neurons after esophageal acid perfusion and the modulation of airway inflammation via the DVC using a guinea pig model of GER.

## Materials and Methods

### Animals

**FIGURE 1:**
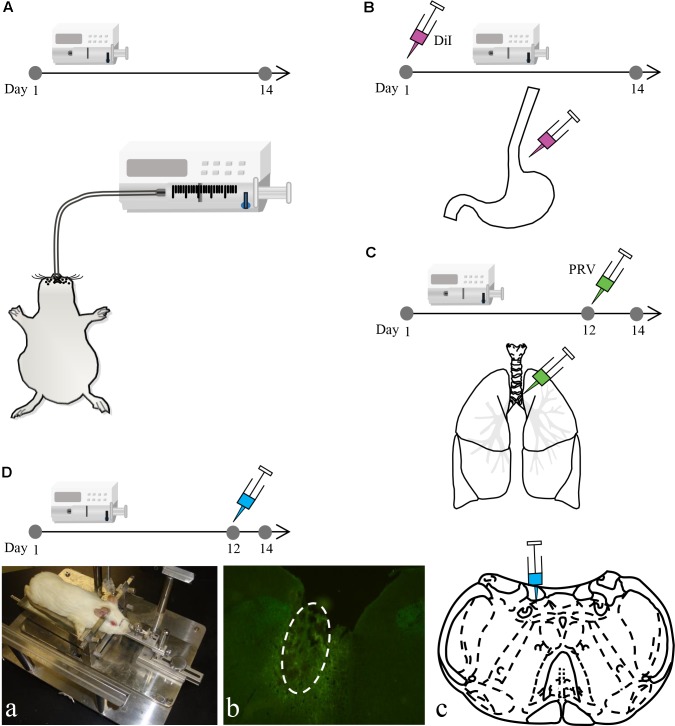
The experimental process of the study. **(A)** Intra-esophageal perfusion (saline, sham and HCl group) of guinea pigs for 14 consecutive days. **(B)** DiI injection into the lower esophageal wall on the 1st day in guinea pigs in the HCl group. **(C)** PRV injection into the tracheal wall on the 12th day in guinea pigs in the HCl group. **(D)** Microinjection into the DVC on the 12th day in guinea pigs in the HCl and saline group. The animal was fixed on a stereotaxic instrument **(a)**. The microinjection site (white circle) and coronal schematic are presented in **(b,c)**.

Male albino Hartley guinea pigs (*n* = 156 in total, body weight: 350–400 g) were purchased from the Experimental Animal Center of Jiangsu Province. The Animal Research Committee of Guangzhou Medical University and Southeast University approved the study protocol. Similar to previously described methods (Liu et al., 2013), acid perfusion was performed by anesthetizing guinea pigs in the HCl group (*n* = 78) with ketamine hydrochloride [50 mg/kg, intraperitoneally (i.p.)] and then perfusing with 0.1 mol/l HCl (including 0.5% pepsin) into the lower esophagus (8 drops/min, 20 min/day) via a stomach tube once per day for 14 consecutive days. Guinea pigs in the saline group (*n* = 66) were perfused with saline, whereas those in the sham group (*n* = 6) only had a stomach tube inserted. Control guinea pigs (*n* = 6) were fed normally (**Figure 1A**).

### DiI Tracing

Six guinea pigs perfused with HCl for 14 consecutive days were anesthetized with ketamine hydrochloride (50 mg/kg, i.p.) on the 1st day for DiI tracing (**Figure 1B**). All four limbs were restrained, and the abdominal skin was disinfected. The upper abdominal muscles were dissected to expose the stomach and lower esophagus. The lower esophagus was then separated. A microsyringe (Nanofil NF34BV-2; WPI) was used to inject a total of 10 μl of 5% DiI (Sigma-Aldrich) into the esophageal wall. The needle remained in place for 1 min after the injection was complete to prevent liquid overflow. Finally, the abdominal muscles and skin were cleaned and sutured. When the animals recovered, they were returned to their cages for feeding.

### PRV Retrograde Tracing

Six guinea pigs that were perfused with HCl for 14 consecutive days were anesthetized with ketamine hydrochloride (50 mg/kg, i.p.) on the 12th day for PRV tracing (**Figure 1C**). All four limbs were restrained, and the skin on the neck was disinfected. The neck muscles were dissected to expose the esophagus and trachea. The trachea was then separated. A microsyringe (Nanofil NF34BV-2; WPI) was used to inject a total of 10 μl of pseudorabies virus Bartha (PRV-Bartha) (10^5^ pfu/ml) into trachea wall sites. The needle remained in place for 1 min after the injection was complete to prevent liquid overflow. Finally, the neck muscles and skin were cleaned and sutured. When the animals recovered, they were returned to their cages for feeding.

### Nuclei Microinjection

A total of sixty guinea pigs in the HCl perfusion group were anesthetized with pentobarbital (30 mg/kg, i.p.) on the 12th day. Prior to surgery, guinea pigs were placed on a warming pad and fixed to a stereotaxic apparatus (**Figure 1D**). The microinjection coordinates were determined based on Canning’s study (Canning and Mori, 2010). Guinea pigs were randomly divided into six groups (*n* = 10 per group), and each group was microinjected with one of the six following agents: artificial cerebrospinal fluid (ACSF; 0.5 μl) to simulate a normal environment, glutamic acid (Glu; 2 mg/ml, 0.5 μl) to excite neurons, γ-aminobutyric acid (GABA; 5 mg/ml, 0.5 μl) to inhibit neurons, lidocaine (2%, 0.5 μl) to induce palsy, exogenous SP (5 mg/ml, 0.5 μl) to increase the concentration of SP in the target area and anti-SP serum (1:200, 0.5 μl) to neutralize SP in the target area. The volume was injected over 5 min; the needle was not removed for an additional 5 min to prevent liquid overflow. Finally, the skin on the scalp was cleaned and sutured. To evaluate the effect of the drugs on control animals, the nuclei microinjection was also performed in the additional sixty guinea pigs with saline perfusion group, and the operation and drugs were described above.

### Evans Blue Dye (EBD) Detection for Airway Microvascular Leakage

Evans blue dye was injected after the last acid perfusion in five randomly selected animals in each group that received nuclei microinjection. As previously reported, leakage reflects vasodilatation and inflammation (Saria and Lundberg, 1983). EBD (30 mg/kg) was injected into the left internal jugular vein after the last HCl perfusion. Animals were anesthetized with pentobarbitone (30 mg/kg, i.p.) and transcardially perfused with 100 ml 0.9% saline to exclude EBD from the blood vessels. Trachea and bronchi were separated from the lungs and dried with filter paper. Then, parts of the trachea and bronchi were coronally sectioned into six 10-μm pieces that were observed using an Olympus fluorescence microscope. The other tissues were weighed and placed in methanamide at 37°C for 24 h to extract the EBD. Absorbance was measured with a spectrophotometer (wavelength 620 nm). The EBD concentration was calculated based on the EBD standard curve (0.5–10 μg/ml range).

### Specimen Processing

On the 14th day, animals were anesthetized with pentobarbitone (30 mg/kg, i.p.).

The control, saline, sham and HCl group (*n* = 6 per group) animals were transcardially perfused with 0.3% phosphate buffered saline (PBS). Then, the left lung and bronchial tissues were rapidly removed (for ELISA), and the animals were then perfused with 4% paraformaldehyde in PBS. The brain, right lung, and bronchia were removed, placed in 4% paraformaldehyde at 4°C for 4 h and then cryoprotected in 30% sucrose at 4°C overnight. Parts of right lung and bronchia were embedded in paraffin. The tissue was then sectioned at 5 μm for hematoxylin and eosin (HE) staining and observed using an Olympus light microscope. The remaining right lung and brainstem tissues were frozen with OCT and coronally sectioned at 20 μm (lung tissues at 40 μm) using a Leica freezing microtome for immunohistochemistry and immunofluorescence, respectively. The total brainstem sections were 2 mm thick (1 mm from rostral and caudal to obex, separately). One section was selected from every five consecutive sections for immunofluorescence and immunohistochemistry.

Brains from DiI tracing and PRV tracing animals (*n* = 6, separately) were removed as described above. DiI was observed directly using an Olympus fluorescence microscope, and PRV was observed by immunofluorescence.

The lungs and bronchial tissues of other animals receiving microinjection (*n* = 30) without EBD injection were removed as described above. Left lungs and bronchial tissues were used for ELISA, and the right tissues were used for immunohistochemistry.

### Immunohistochemistry

Lung tissue sections were incubated with 3% H_2_O_2_ for 15 min to block endogenous peroxidase activity, washed with 0.3% PBS (3 × 5 min), incubated for 1 h at room temperature with a blocking solution (10% goat serum), and subsequently incubated overnight with the primary antibody (mouse anti-SP; 1:200; Abcam). The tissue was washed with 0.3% PBS (3 × 5 min) followed by incubation for 1 h at room temperature with a biotinylated second antibody (goat anti-mouse; 1:300; Abcam). After washing with 0.3% PBS (3 × 5 min), sections were incubated for 30 min with avidin/biotinylated horseradish peroxidase (HRP), washed with 0.3% PBS (3 × 5 min), and reacted with DAB as a chromogen. Sections were observed using an Olympus light microscope.

### Immunofluorescence

Brainstem sections were washed with 0.3% PBS and 0.4% Triton-X 100 (3 × 5 min) and incubated for 1 h at room temperature with a blocking solution (10% goat serum), then overnight with primary antibodies (rabbit anti-Fos, 1:500, Santa Cruz; mouse anti-SP, 1:200 Abcam; and rabbit anti-PRV, 1:200, Abcam). Sections were then washed with PBS (3 × 5 min) followed by incubation for 1 h at room temperature with the appropriate secondary antibodies (AlexaFluor^®^ 594-conjugated goat anti-rabbit, 1:400, Invitrogen; AlexaFluor^®^ 488-conjugated goat anti-mouse, 1:400, Invitrogen). After washing (3 × 5 min in PBS), sections were observed using an Olympus fluorescence microscope.

### ELISA

The bronchi and lungs were weighed, boiled (100°C) for 10 min in 1 M acetic acid (1:10, wt/vol), diluted with 0.1 M PBS and homogenized. Homogenates were transferred to polypropylene tubes and centrifuged (40,000 ×*g*, 4°C, 20 min). Before measurements were taken, the supernatant was centrifuged again (40,000 × g, 4°C, 20 min). The SP concentration was measured using an ELISA kit (Cayman) following the manufacturer’s instructions.

### Statistical Analysis

Data were expressed as the mean ± SD and analyzed for significant differences using SPSS 17.0 software. Comparisons among multiple groups were performed using one-way analysis of variance (ANOVA). A *p*-value < 0.05 was considered statistically significant. The mean densities for immunohistochemistry and EBD were determined using Image-Pro Plus (IPP). Immunofluorescence measurements were determined at the same time that positive neurons were counted using IPP. Six lung and brain tissue sections each were selected randomly for statistical analysis.

## Results

### Inflammation in the Airway and Esophagus After Repeated Intra-Esophageal HCl Perfusion

**FIGURE 2:**
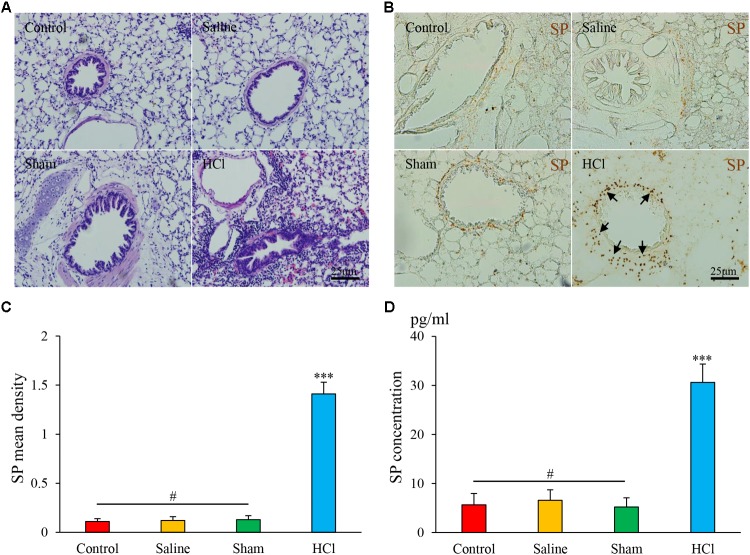
HCl perfusion into the esophagus caused airway inflammation. **(A)** Pathological changes in the lung tissues of guinea pigs in four groups as assessed by HE staining. **(B)** SP expression (black arrow) in the airway of the HCl group animals was increased compared with that of the other three groups. **(C)** SP mean density is presented. **(D)** SP concentrations were measured by ELISA. Data are expressed as the mean ± SD. ^∗∗∗^*p* < 0.001 compared with the other three groups; ^#^*p* > 0.05 among the control, saline and sham groups; one-way ANOVA followed by LSD test.

In the HCl-treated group, we observed tracheal and bronchial mucosal edema, partial epithelial shedding, vasodilation, and mucosa and submucosa congestion (**Figure 2A**). No abnormalities in the tracheal mucosa were observed in the other three groups. We also excluded HCl direct reflux or microaspiration into the airway by performing 99mTc tracing as described in our previous study (Liu et al., 2013).

### Esophageal HCl Perfusion Increased SP Expression in the Airway

SP-like immunoreactivity (SP-li) was observed in bronchus and lung tissues, predominantly in the cytoplasm of epithelial cells in the peribronchial region (**Figure 2B**). The mean density of SP-li staining was significantly increased in the HCl-treated group (1.41 ± 0.12, **Figure 2C**) compared with the other groups, and the SP concentration was increased in the HCl group (30.60 ± 3.73, **Figure 2D**). No significant differences were detected among the other three groups. These data demonstrate that intra-esophageal HCl perfusion induced the release of neurogenic inflammatory mediators in the airway.

### Intra-Esophageal HCl Perfusion Enhanced Neuronal Activities and SP Expression in the DVC

**FIGURE 3:**
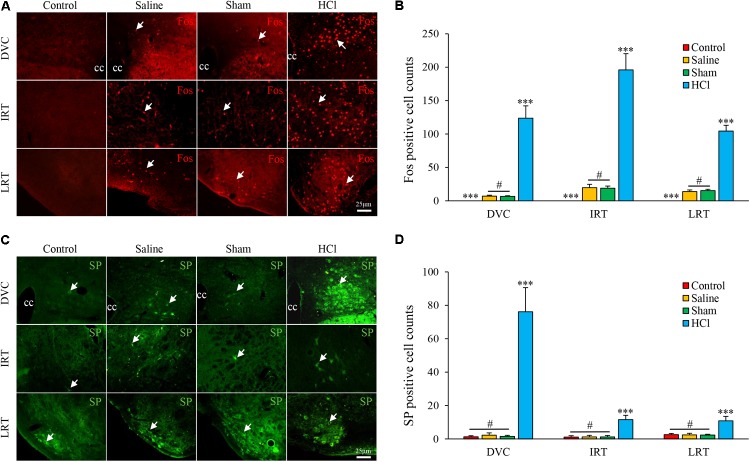
Fos and SP expression in the nuclei of the medulla oblongata in four groups. **(A)** Fos expression (white arrow) in the DVC, IRT, and LRT in the HCl group was increased compared with expression in the other three groups. Fos-positive cells were not observed in the control group. **(B)** Fos-positive cell counts are presented. **(C)** SP expression (white arrow) in the DVC, IRT, and LRT was increased in the HCl group compared with expression in the other three groups. **(D)** SP-positive cell counts are presented. Data are expressed as the mean ± SD. ^∗∗∗^*p* < 0.001 compared with the other three groups; ^#^*p* > 0.05 for Fos-positive cell counts between the saline and sham groups; ^#^*p* > 0.05 for SP-positive cell counts among the control, saline and sham groups; Dunnett’s T3 test. cc, central canal.

Fos-positive cells were primarily distributed in the DVC [123.75 ± 18.42, including the NTS and dorsal motor nucleus of the vagus (DMV)], intermediate reticular nucleus (IRT, 195.86 ± 24.39), and lateral reticular nucleus (LRT, 104.39 ± 8.70) in the HCl-treated group. No Fos-positive cells were observed in the control group. No differences were noted between the saline and sham groups (**Figures 3A,B**).

SP-positive cells were located in the cytoplasm and primarily distributed in the DVC (76.17 ± 14.45), IRT (11.64 ± 2.56), and LRT (10.86 ± 2.63) in the HCl-treated group. Few SP-positive neurons were observed in other groups, and no significant differences in SP expression were noted among the other three groups (**Figures 3C,D**).

### Co-expression of Fos and SP in the DVC of GER Guinea Pigs

**FIGURE 4:**
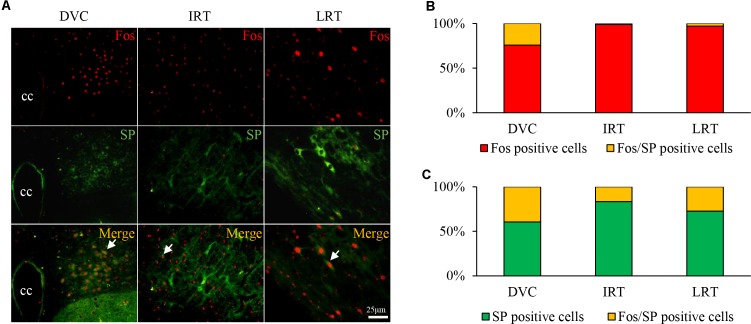
Fos/SP-positive cell distribution in the nuclei of the medulla oblongata in the HCl group. **(A)** Fos/SP-positive cells (white arrow) were primarily distributed in the DVC (including NTS and DMV), and a few cells were observed in the IRT and LRT. **(B,C)** Fos/SP-positive cells represent a large proportion of Fos- or SP-positive cells, especially in the DVC.

A large number of Fos/SP double-labeled cells were primarily observed in the DVC of HCl-treated guinea pigs (**Figure 4A**), accounting for approximately 24.19% of Fos-positive cells and 39.47% of SP-positive cells observed (**Figures 4B,C**). Double-labeled neurons were occasionally observed in the IRT and LRT. The results showed that HCl perfusion into the lower esophagus caused neuron activity and SP expression.

### DiI Tracing Between the Esophagus and Medulla Oblongata

**FIGURE 5:**
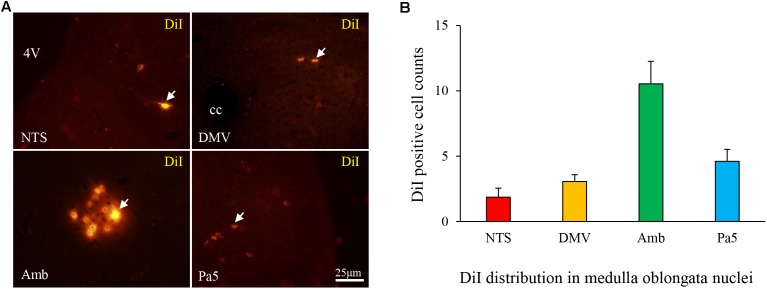
**(A)** DiI-labeled cells (white arrow) were observed in the nuclei of the medulla oblongata in the HCl group and primarily distributed in the Amb **(B)**.

DiI-labeled neurons were distributed in the NTS (1.86 ± 0.69), DMV (3.06 ± 0.52), nucleus ambiguous (Amb, 10.53 ± 1.72), and Pa5 (4.61 ± 0.92) (**Figures 5A,B**). These findings proved the connection between the lower esophagus and nuclei in the medulla oblongata.

### Neural Connections to the Airway Were Identified by PRV Tracing in GER Guinea Pigs

**FIGURE 6:**
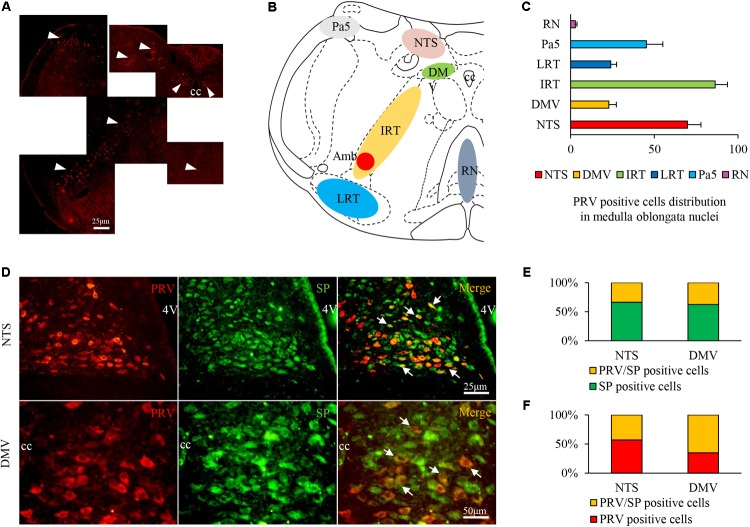
PRV/SP-positive cell distribution in the nuclei of the medulla oblongata in the HCl group. **(A)** PRV-infected cells were located in most nuclei of the medulla oblongata (white triangle). **(B)** PRV-infected cell distribution in the coronal schematic of the medulla oblongata. **(C)** PRV-positive cell counts in different nuclei. **(D)** PRV/SP-positive cells (white arrow) were primarily located in the NTS and DMV. **(E)** The ratio of PRV/SP-positive cells among SP-positive cells. **(F)** The ratio of PRV/SP-positive cells among PRV-positive cells was quite high. 4V, fourth ventricle.

Pseudorabies virus-infected neurons were observed in multiple nuclei, including the NTS (69.78 ± 8.06), DMV (22.83 ± 4.45), paratrigeminal nucleus (Pa5, 45.31 ± 9.75), IRT (86.31 ± 7.36), LRT (24.03 ± 3.47), and raphe nuclei (RN, 2.89 ± 0.93) (**Figures 6A–C**). Most PRV/SP double-labeled neurons were distributed in the NTS (approximately 44.68% of PRV single-labeled neurons and 33.87% of SP single-labeled neurons) and DMV (65.22% of PRV single-labeled neurons and 37.50% of SP single-labeled neurons), with few observed in the other nuclei (**Figures 6D–F**). The results indicated that the NTS and DMV neurons connecting to the airway were active, and SP expression increased during HCl intra-esophageal perfusion.

### Airway Inflammation Is Modulated via the DVC

**FIGURE 7:**
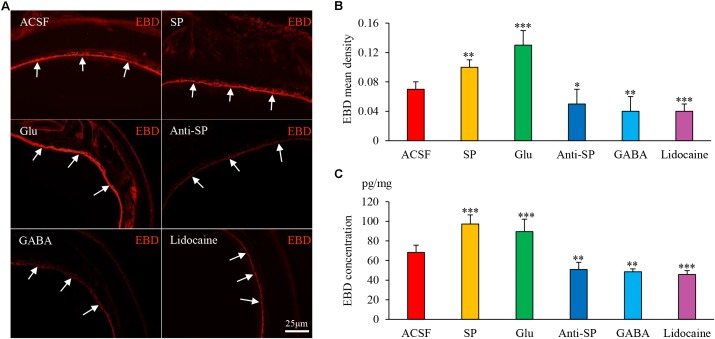
The effects of microinjection into the DVC on airway microvascular leakage in a guinea pig model of HCl perfusion. Airway inflammation was aggravated by microinjection of exogenous SP and Glu into the DVC but was alleviated by anti-SP serum, GABA, and lidocaine. **(A)** Airway microvascular leakage was observed via EBD (white arrow) after drug microinjection into the DVC. EBD mean densities and concentrations in six groups are presented in **(B,C)**. Data are expressed as the mean ± SD. ^∗^*p* < 0.05, ^∗∗^*p* < 0.01, and ^∗∗∗^*p* < 0.001 *vs* ACSF group; one-way ANOVA followed by LSD test.

**FIGURE 8:**
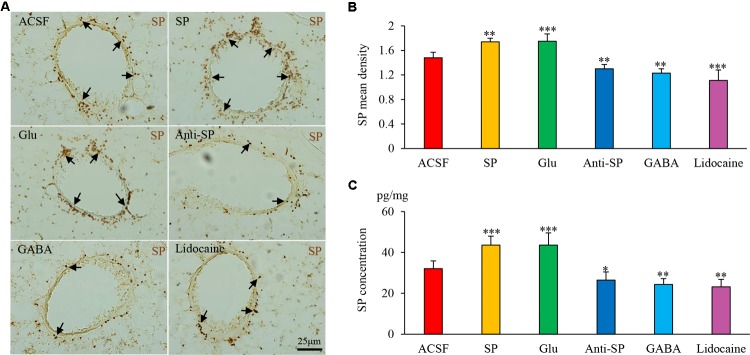
The effects of microinjection into the DVC on SP airway expression in a guinea pig model with HCl perfusion. **(A)** SP expression (brown staining, black arrows) in the airway after drug microinjection into the DVC. SP mean densities and concentrations (measured by ELISA) in six groups are presented in **(B,C)**. Data are expressed as the mean ± SD. ^∗^*p* < 0.05, ^∗∗^*p* < 0.01, and ^∗∗∗^*p* < 0.001 *vs* ACSF group; one-way ANOVA followed by LSD test.

**FIGURE 9:**
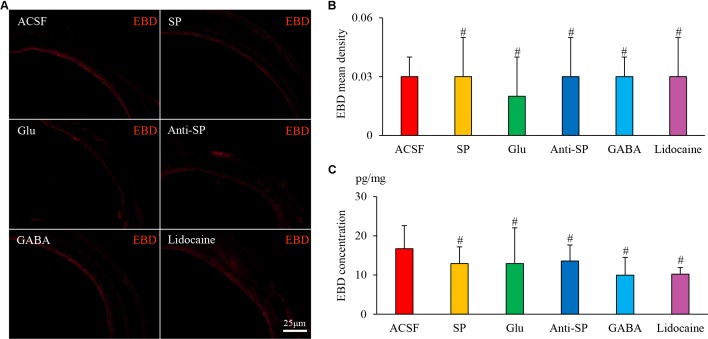
The effects of microinjection into the DVC on airway microvascular leakage in a guinea pig model of saline perfusion. **(A)** Airway microvascular leakage was observed via EBD after drug microinjection into the DVC. EBD mean densities and concentrations in six groups are presented in **(B,C)**. Data are expressed as the mean ± SD. ^#^*p* > 0.05 *vs* ACSF group; one-way ANOVA followed by LSD test.

**FIGURE 10:**
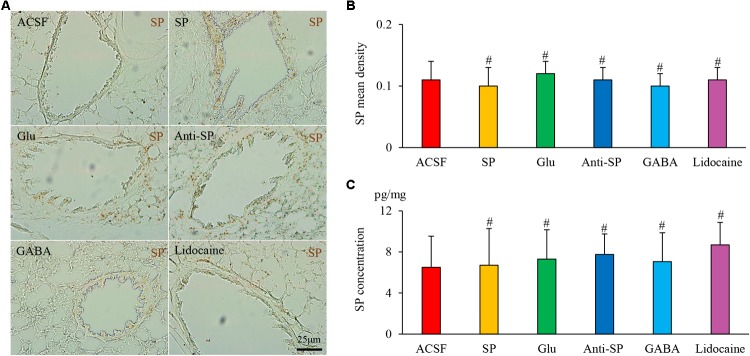
The effects of microinjection into the DVC on SP airway expression in a guinea pig model with saline perfusion. **(A)** SP expression in the airway after drug microinjection into the DVC. SP mean densities and concentrations in six groups are presented in **(B,C)**. Data are expressed as the mean ± SD. ^#^*p* > 0.05 *vs* ACSF group; one-way ANOVA followed by LSD test.

Microinjection was performed to alter neuronal activities and SP concentrations, and SP expression and microvascular leakage in the airway were subsequently assessed in the HCl group (**Figures 7**, **8**). Glu or exogenous SP significantly increased airway microvascular leakage (EBD mean density, Glu 0.13 ± 0.02 and exogenous SP 0.10 ± 0.01 *vs* ACSF 0.07 ± 0.01; EBD concentration, Glu 89.50 ± 12.73 and exogenous SP 97.34 ± 9.22 *vs* ACSF 68.22 ± 7.44) and SP expression (SP mean density, Glu 1.75 ± 0.12 and exogenous SP 1.74 ± 0.06 *vs* ACSF 1.48 ± 0.09; SP concentration, Glu 43.58 ± 6.00 and exogenous SP 43.58 ± 4.41 *vs* ACSF 32.06 ± 3.83). Opposite results were observed following the injection of anti-SP serum, GABA, or lidocaine (EBD mean density, anti-SP 0.05 ± 0.02, GABA 0.04 ± 0.02 and lidocaine 0.04 ± 0.01 *vs* ACSF 0.07 ± 0.01; EBD concentration, anti-SP 50.81 ± 7.32, GABA 48.56 ± 2.92 and lidocaine 45.72 ± 3.95 *vs* ACSF 68.22 ± 7.44) and SP expression (SP mean density, anti-SP 1.30 ± 0.07, GABA 1.23 ± 0.07 and lidocaine 1.11 ± 0.17 *vs* ACSF 0.07 ± 0.01; SP concentration, anti-SP 26.44 ± 4.03, GABA 24.33 ± 2.82 and lidocaine 23.16 ± 3.62 *vs* ACSF 68.22 ± 7.44). In the saline perfusion animals, there were no differences in the SP and EBD densities and concentrations among all the six groups (**Figures 9**, **10**). These results revealed that airway neurogenic inflammation was altered by DVC neuronal activities and SP concentration in esophageal HCl perfusion.

## Discussion

Many studies have shown associations between airway inflammation and GER, but the brainstem nuclei involved in the progression of airway inflammation is still uncertain. In this study, we have found DVC neurons may modulate neurogenic airway inflammation in guinea pigs with esophageal HCl perfusion.

Previous studies (Gestreau et al., 1997; Ohi et al., 2005; Jakus et al., 2008) have confirmed the location of neurons related to cough using *c-fos*. Jakus et al. (2008) used Fos to locate the brainstem neurons related to cough and revealed that a large number of the medulla oblongata, pons, and midbrain neural nuclei are involved in the regulation of coughing in cats. The central terminals of cough receptors are a critical component to cough gating, and terminals localized in the medial subnuclei of NTS were confirmed by microinjection and dual-tracing studies (Canning and Mori, 2010). In this study, airway inflammation was influenced by the microinjection of Glu, GABA, or lidocaine, which altered neuronal activity in the injected areas. Our findings revealed that the DVC potentially modulates airway inflammation related to GER.

It is clear that SP increases the excitability of nucleus of the NTS neurons, thereby facilitating lung afferent transmission in guinea pigs and rabbits (Mutoh et al., 2000; Mutolo et al., 2007). SP is also closely associated with the inflammation observed in respiratory diseases. In this study, intra-esophageal HCl perfusion induced SP expression in the lung and CNS, and SP-immunoreactive nerve terminals were abundant in the DVC, particularly in the NTS. SP expression was increased in the NTS and DMV, where many Fos/SP double-labeled neurons were observed in GER guinea pigs. These findings indicate that Fos/SP-positive neurons were active, and SP was localized in the soma, raising questions about the relationship between central and peripheral SP. Our microinjection results reveal that airway inflammation, vasodilation, and plasma leakage were also consistent with changes in SP levels in the DVC following the injection of SP or anti-SP serum. We hypothesize that neuronal excitability or SP levels in the DVC influence nerve endings that innervate the airway, subsequently increasing SP release.

Higher brain circuitry was involved in the processing of respiratory sensations according to our neural tracing results, suggesting that sensations arising from airway vagal afferent projections ascend the neuraxis through multiple hindbrain and subcortical nuclei to provide sensory input to the cerebral cortex (McGovern et al., 2012a,b, 2015). In this experiment, we employed PRV-Bartha, a well-defined retrograde tracer used to study the pathway from the airway to the medulla oblongata. The location and number of PRV-infected neurons may not be accurate, particularly for PRV-infected and SP-positive cells, due to the neurotoxicity of PRV. As PRV spreads to synapse-linked neurons, our results reveal that the trachea wall is innervated by neurons of the NTS and DMV; most PRV/SP double-labeled neurons were located in the NTS and DMV. In combination with our microinjection results, we conclude that the NTS and DMV may regulate airway inflammation.

**FIGURE 11:**
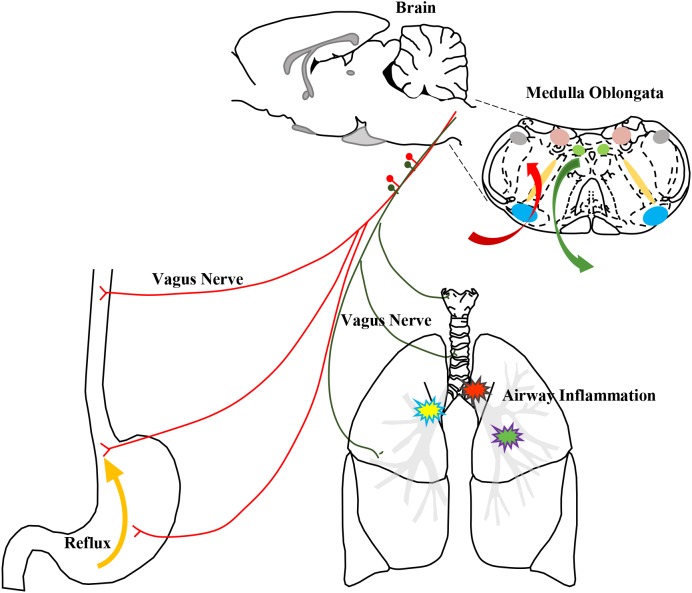
The schematic of airway neurogenic inflammation induced by HCl intra-esophageal perfusion regulated by the DVC. The stimulation of HCl reflux into the lower esophagus was input via the vagus nerve to the nuclei in the medulla oblongata, especially NTS and DMV, and the integrated information was output via the vagus nerve into the airway to induce changes in airway neurogenic inflammation.

The NTS is a sensory nucleus, a visceral sensation center that receives input from afferent nerves. Brain activities that regulate the lung and airway enhance neurogenic inflammation induced by neuropeptides given that CNS activities “enlarge” lung inflammation during asthma attacks (Mazzone and Canning, 2002; Widdicombe, 2003). The vagus nerve, which is an important pathway of communication from the immune system to the brain, transmits inflammatory information to the CNS (Banks and Kastin, 1991; Dantzer et al., 2000; Dantzer and Wollman, 2003; Wrona, 2006), and inflammation is also modulated by the vagus nerve and reflex (Tracey, 2002; Pavlov and Tracey, 2012). In this GER model, the CNS may exhibit a “pathological status”, and CNS inflammation may influence the airway mutually. Similar to a report by Mazzone describing communication from the esophagus to the nuclei in the medulla oblongata detected by DiI tracing (McGovern and Mazzone, 2010), the DVC receives afferent information from the esophagus and thereby controls peripheral airway inflammation in our models. PRV-labeled neurons were observed in the NTS and DMV, revealing communication between these sites and the Amb, which innervates motor fibers. Based on our results, GER associated airway inflammation and cough mediated by esophageal–bronchial reflex arc are involved in DVC. Gastric contents stimulate the receptors of the lower esophagus, and afferent signals are transmitted via vagal afferent fibers to the DVC, which is the primary center of the reflex arc. The excited impulses from the active SP neurons are conducted to the airway innervated by SP nerve fibers, which are important components of vagal efferent nerves. The activated SP fibers release SP to enhance inflammatory mediators and cough sensitivity, thereby causing cough (**Figure 11**). This action forms the vago-vagal reflex between the esophagus and airway. Airway neurogenic inflammation is associated with not only local airway inflammation but also brain neuron activity. The CNS neurons play an important role in the pathological processes of inflammation and enhanced cough sensitivity.

Chronic cough may be treated as a neuropathic disorder (Chung et al., 2013). Gabapentin (GABA receptor agonist) and baclofen (GABA analogue) exhibit satisfactory treatment results in some patients with refractory cough. It is also suggested that CNS neurons may be involved in postinfectious cough that responds poorly to standard treatments. For further treatment of GER-associated cough, chronic refractory cough, and even severe asthma, the CNS may serve as a therapeutic target, and blocking the CNS to alleviate airway neurogenic inflammation may provide insight for future drug development.

In conclusion, the DVC is involved in the esophageal-bronchial reflex and modulates neurogenic airway inflammation induced by GER, and it maybe a possible mechanism of airway neurogenic inflammation related to GER.

## Ethics Statement

This study was carried out in accordance with the recommendations of the Guide for the Care and Use of Laboratory Animals published by the US National Institutes of Health. The protocol was approved by the Animal Research Committee of Guangzhou Medical University and Southeast University.

## Author Contributions

KL and RD contributed to the study design. ZC contributed to the preparation of GER models and manuscript preparation. LS, HC, ZY, and TP contributed to PRV retrograde tracing. HC and DG contributed to microinjection and DiI tracing. WZ contributed to PRV retrograde tracing, data collection, and analysis.

## Conflict of Interest Statement

The authors declare that the research was conducted in the absence of any commercial or financial relationships that could be construed as a potential conflict of interest.
